# Cadmium-Induced Oxidative Stress Impairs Glycemic Control in Adolescents

**DOI:** 10.1155/2017/6341671

**Published:** 2017-12-12

**Authors:** Gabriele Pizzino, Natasha Irrera, Alessandra Bitto, Giovanni Pallio, Federica Mannino, Vincenzo Arcoraci, Federica Aliquò, Letteria Minutoli, Chiara De Ponte, Paola D'andrea, Francesco Squadrito, Domenica Altavilla

**Affiliations:** ^1^Department of Clinical and Experimental Medicine, University of Messina, Messina, Italy; ^2^Department of Pharmacy, University of Bari, Bari, Italy; ^3^Department of Biomedical, Dental, Morphological and Functional Sciences, University of Messina, Messina, Italy

## Abstract

Experimental evidence suggests that cadmium (Cd) boosts oxidative stress that may result in toxicity on the endocrine system also in humans. The aim of this study was to investigate the glycemic control and oxidative stress markers in male adolescents with increased urinary levels of cadmium. We investigated 111 males, aged 12–14 years, living in a polluted area of Sicily and a control age-matched population (*n* = 60) living 28–45 km far from the polluted site. Malondialdehyde (MDA), total antioxidant activity (TAC), metallothionein-1A (MT-1A) gene expression, insulin resistance by the homeostatic model assessment (HOMA-IR), and urinary cadmium were investigated. Cd levels were significantly higher in adolescents living in the polluted area than in control age-matched subjects. Adolescents with elevated Cd levels had a significant increase in MDA, MT-1A, and HOMA-IR and reduced TAC compared to the control group. A robust correlation was found between urinary cadmium and MT-1A, HOMA-IR, and MDA whereas an inverse correlation was identified between urinary cadmium and TAC. This study indicates that cadmium burden alters glycemic control in adolescents and suggests that oxidative stress plays a key role in cadmium-induced insulin resistance, increasing the risk of developing metabolic disorders.

## 1. Introduction

Air pollution represents an important risk factor for the development of diabetes and obesity. Air pollution, in fact, boosts a robust oxidative stress and a sustained activation of the inflammatory cascade that in turn causes lipogenesis, adipose tissue inflammation, and insulin resistance [1, 2]. Interestingly, ambient air pollution is also associated with predisposing conditions for diabetes and insulin resistance. Particulate matter (PM), a complex mixture of small particles of various sizes (ranging from 10 *μ*m to 0.2 *μ*m) formed by both numerous components (including nitrates, sulfates, organic chemicals, and heavy metals) and liquid droplets, is used currently to monitor outdoor air pollution. It has been shown that higher PM_10_ (particulate matter of 10 micrometers or less) levels were correlated with higher concentration of HbA_1c_ in Germans affected by type 2 diabetes (T2D), which may be considered as a read-out of the average glycemia during the previous 30–120 days [3]. Several pollutants were also reported to display negative effects on HbA_1c_ and fasting glucose levels in an elderly Taiwanese population [4]. A Korean study showed that subjects with a history of diabetes or with diabetes, exposed to nitric oxide (NO_2_), had augmented homeostatic model assessment of insulin resistance (HOMA-IR) [5]. In agreement with these findings, it has been reported that healthy adults living in a nonpolluted area showed decreased insulin sensitivity after they were exposed to air pollution in a heavy-traffic urban area for 4-5 hours each day for five consecutive days [6]. Ambient air pollution also alters insulin resistance in healthy children and adolescents: exposure to NO_2_ and PM_10_ was, in fact, correlated with increased HOMA-IR in German adolescents [7]. Furthermore, HOMA-IR was augmented in 374 Iranian children aged 10–18 years following short-term coexposure to carbon monoxide (CO) and PM_10_ [8].

Generally speaking, there are poor data concerning children; this generates concerns. Indeed, children may absorb heavy metals, including Cd, more readily than adults and they are more susceptible to its toxicity because of biologic and developmental reasons. Cadmium (Cd) is an endocrine disruptor that interferes with metabolic homeostasis and normal development [9–11]. It is produced by the emission of the industrial plants, and it is taken up by the ecosystem components, entering the food chain. This explains why, besides the industrial workers, people living near polluted areas may be at enhanced risk of human exposure [12, 13].

The urban area of Milazzo-Valle del Mela (Sicily, Italy) has been indicated to be at high risk of environmental crisis by local government authorities because of the presence of several industrial plants nearby the residential area. We have previously shown that male adolescents living and residing in this area have enhanced urinary Cd levels and show delayed puberty and testis growth [14].

The aim of this study was to investigate insulin resistance by the means of HOMA-IR, a well-known predictor of T2D, and the possible correlation with oxidative stress markers in adolescents living in polluted areas.

## 2. Materials and Methods

### 2.1. Study Design and Population

This study was a part of a cross-sectional investigation aimed to evaluate the correlation between cadmium and pubertal development in adolescents. A population of 111 male children, aged 12–14 years of Caucasian origin, was recruited in the industrial area of Milazzo-Valle del Mela.

A control population (*n* = 60) race, sex, and age matched living 28–45 km far from the industrial site was also enrolled in a volunteer base. The study protocol was adherent with the principles of the Declaration of Helsinki, and all parents or legal tutor participants gave written informed consent. Only subjects of Sicilian origin, healthy and nonsmokers, living in the selected areas from at least 10 years were included in the study.

A medical visit was performed at enrollment, and all children were evaluated by specifically trained nurses and doctors that evaluated the height, weight, body mass index (BMI), and scored pubertal development, according to Tanner classification, as previously reported [14]. Testicular volume was assessed by ultrasound evaluation, and testosterone levels were determined as previously described [14]. A complete family history was obtained and routine evaluations were performed.

### 2.2. Cadmium Urine Levels

All children received urine collection containers for 24 h specimens, and their parents were tutored for apposite procedure and storage. Urines were collected 1 or 2 days before the medical visit and stored at 2–6°C to avoid contamination. Blinded technicians analyzed cadmium urine samples on coded samples using an atomic absorption spectrometer procedure, as previously reported [14].

### 2.3. Determination of Prooxidant Markers, Antioxidant Markers, and HOMA-IR

To assess oxidative stress, concentration of malondialdehyde (MDA), used as a marker of lipid peroxidation index, was measured in plasma using a colorimetric commercial kit (ab118970, Abcam plc, Cambridge, UK). MDA concentration was calculated from a standard curve and expressed as *μ*mol/l.

The total antioxidant capacity (TAC) was determined by the ferric reducing ability of plasma (FRAP) method in which a colourless ferric tripyridyltriazine complex is reduced to a blue ferrous complex by the antioxidants in the serum [15]. Briefly, a mixed solution of 50 *μ*l of serum and 50 *μ*l of distilled water was added to 900 *μ*l of FRAP reagent and incubated at 37°C for 25 min. The change in absorbance at 593 nm is directly related to the total reducing power of electron-donating antioxidants present in the serum. The results were expressed in *μ*mol/dl.

Insulin resistance was assessed using the homeostasis model assessment for insulin resistance (HOMA-IR). HOMA-IR was calculated using the following formula: fasting glucose (mg/dl) × fasting insulin (*μ*IU/ml)/22.5.

### 2.4. Metallothionein (MT-1A) Gene Expression

Total RNA was extracted from whole blood samples (250 *μ*l) using TRIzol reagent (Thermo Fisher Scientific, Waltham, MA, USA), following the manufacturer's protocol, and was quantified with a spectrophotometer (NanoDrop Lite; Thermo Fisher Scientific). Reverse transcription was carried out using 1 *μ*g of RNA by using the SuperScript® VILO™ cDNA synthesis kit (Thermo Fisher Scientific) and random primers, following the manufacturer's protocol. 1 *μ*l of total cDNA was used to quantify MT-1A (catalogue number: Hs00831826_s1), by real-time qPCR, using *β*-actin (catalogue number: 4310881E; Life Technologies) as reference gene. Reactions have been carried out in Singleplex in 96-well plates using the TaqMan Universal PCR master mix and premade hydrolysis probes (Thermo Fisher Scientific). PCR reaction was monitored by using the QuantStudio 6 Flex (Thermo Fisher Scientific), and results were quantified by the 2^−ΔΔCt^ method for both target and reference genes. As calibrator, a nonexposed volunteer was used.

### 2.5. Outcomes

Changes in HOMA-IR, plasma MDA, MT-1A gene expression, and TAC and their dependence to cadmium levels were evaluated.

### 2.6. Statistical Analysis

Standard descriptive statistical analyses were performed to evaluate basal demographic and clinical characteristics. All results were expressed as median with an interquartile range for continue variables, absolute and percentage frequencies for categorical variables.

The Kolmogorov-Smirnov test for normality was used to check data distribution. Because of some not-normal numerical variable, a nonparametric approach was used. The Mann–Whitney *U* test was applied to compare adolescents living in the polluted area and controls with reference to quantitative characteristics.

The nonparametric two-tailed Spearman Rho test was estimated to assess possible associations between all covariates of interest.

Univariate linear regression models were used to assess the possible dependence of HOMA-IR, MDA, MT-1A, and TAC levels by cadmium exposition levels and by each covariate of interest. Moreover, predictors that emerged as significant using the univariate model were included in a multivariate linear regression model.

The nonparametric two-tailed Spearman Rho test was also used to estimate possible interdependence between all the predictors that emerged as significant using the univariate models. This has been done to avoid multicollinearity and, consequently, to better identify the key predictors to include in the multivariate model. Beta coefficient with 95% confidence interval (CI) was calculated for each covariate of interest.

Two-tailed *p* value was set at 0.05 to be considered statistically significant. Statistical analysis was performed by using Statistical Package for Social Science (SPSS Statistics 17.0, Chicago, IL) software.

## 3. Results

The characteristics of population included in the study are reported in Table 1. There were no statistical differences between adolescents living in the polluted area and controls regarding weight, height, and BMI, whereas tanner score, testicular volume, and testosterone were significantly lower in adolescents living in the polluted area. Cadmium urinary levels HOMA-IR and MDA resulted higher in exposed subjects, while TAC was significantly lower with respect to controls (Table 1).

**Figure 1:**
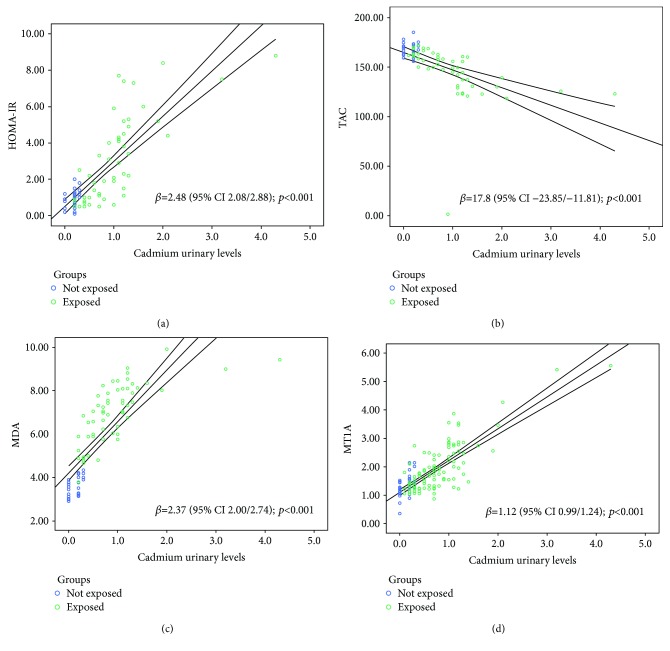
HOMA-IR (a), TAC (b), MDA (c), and MT-1A (d) dependence to cadmium urinary levels in subjects living or not in polluted area: univariate linear regression models.

Cadmium directly influenced HOMA-IR (*β* = 2.48 (95% CI 2.08–2.88); *p* < 0.001; Figure 1(a)) and MDA (*β* = 2.37 (95% CI 2.00–2.74); *p* < 0.001; Figure 1(b)), as assessed by the univariate linear regression models. On the contrary, TAC was inversely related to urinary cadmium levels (*β* = −17.8 (95% CI −23.85/−11.81); *p* < 0.001; Figure 1(c)).

Instead, HOMA-IR was inversely related to Tanner stage (*β* = −0.90 (95% CI −1.40/−0.40); *p* = 0.001), as well as testicular volume (*β* = −0.19 (95% CI −0.28/−0.09); *p* < 0.001), and testosterone levels (*β* = −0.13 (95% CI −0.20/−0.06); *p* < 0.001). On the contrary, TAC was directly influenced by testicular volume (*β* = 1.22 (95% CI 0.10–2.35); *p* = 0.033) and testosterone levels (*β* = 1.18 (95% CI 0.41–1.96); *p* = 0.003).

MDA was inversely related to Tanner stage (*β* = −1.09 (95% CI −1.47/−0.71); *p* < 0.001), testicular volume (*β* = −0.25 (95% CI −0.33/−0.17); *p* < 0.001), and testosterone levels (*β* = −0.18 (95% CI −0.23/−0.13); *p* < 0.001). On the contrary, age directly influenced MDA (*β* = 0.99 [95% CI 0.38–1.61]; *p* = 0.002).

The expression of the MT-1A gene coding for metallothionein (isoform 1A) was directly related to cadmium levels (*β* = 1.12 (95% CI 0.99/1.24); *p* < 0.001; Figure 1(d)) and inversely related to testis volume (*β* = −0.04 (95% CI −0.07/−0.01); *p* = 0.005), in the univariate model.

Because of interdependence between cadmium and Tanner stage (*r*_s_ −346; *p* < 0.001), testicular volume (*r*_s_ −456; *p* < 0.001), and testosterone levels (*r*_s_ −635; *p* < 0.001); and testicular volume and age (*r*_s_ 243; *p* < 0.001), Tanner stage (*r*_s_ 914; *p* < 0.001), and testosterone levels (*r*_s_ 755; *p* < 0.001), only cadmium and testicular volume were included in the multivariate linear regression model

Cadmium urinary levels resulted the only key predictors influencing HOMA-IR (*β* = 2.41 (95% CI 1.97–2.85); *p* < 0.001), TAC (*β* = −17.81 (95% CI −4.5/−11.12); *p* < 0.001), and MT-1A (*β* = 1.16 (95% CI 1.03/1.29); *p* < 0.001); whereas, cadmium urinary levels (*β* = 2.03 (95% CI 1.65–2.41); *p* < 0.001) and testicular volume (*β* = −0.12 (95% CI −0.17/−0.06); *p* < 0.001) are independent key predictors influencing MDA.

## 4. Discussion

The hyperglycemic potential of Cd has been demonstrated in both animal models and humans. Systemic cadmium exposure causes marked changes on several parameters of glycemic metabolism that are abated by the administration of estradiol [16] and most interestingly by antioxidants [17].

In vitro, Cd also impairs insulin secretion by pancreatic beta cells and this is associated with an increase in metallothioneins, the most important cadmium-binding proteins [18]. As a matter of fact, the gene expression of metallothioneins increases following Cd exposure in adults and it has been related to renal toxicity [19]. The present data support these previous findings; in fact, the gene expression of MT-1A was significantly increased in Cd-exposed adolescents as compared to controls. In addition, this increase was related to a reduced testicular volume suggesting that cadmium not only reduces testis growth in adolescents but is also responsible for an increase in MT-1A as a compensatory detoxification mechanism.

An epidemiological study confirmed the Cd-disrupting effect on glycemic control: in fact, enhanced blood glucose level and reduced serum insulin levels were reported in smelter workers exposed to cadmium [20]. All these findings, taken together, strongly support the potential of Cd to cause insulin resistance, a predisposing condition for developing diabetes. Indeed, the adolescents exposed to cadmium revealed an altered insulin resistance that may actually predispose to diabetes. In addition, HOMA-IR also correlated with MDA and TAC variations that strongly indicates some level of Cd-induced systemic toxicity. However, as far as we know, no study has investigated the mechanisms underlying this Cd effect in humans and especially in adolescents.

Taking in consideration the results obtained so far, monitoring the internal exposure to chemicals in biological fluids (human biomonitoring (HBM)) may be useful to study the possible effects of chronic low environmental exposure to pollutants in the general population of industrialized countries, especially those particularly susceptible such as adolescents. Using this methodology, we have shown that adolescents, living in the industrialized area of Milazzo-Valle del Mela in the north of Sicily, have increased urinary cadmium levels [21] that are robustly associated to a marked oxidative stress [22] and to a delayed puberty onset and testicular weight in males [14].

Considering the ability of cadmium to generate insulin resistance and reactive oxygen species, we investigated on a possible correlation between the heavy metal and these two variables. Our results suggest that adolescents with higher urinary levels of Cd had increased HOMA-IR and plasma malondialdehyde together with reduced total antioxidant activity. A robust correlation was also found between urinary cadmium and both HOMA-IR and MDA, whereas an inverse correlation was identified between urinary cadmium and TAC. As far as we know, this is the first study demonstrating a correlation between increased internal exposure of Cd and elevated insulin resistance. Furthermore, this study confirms the mechanistic role of oxidative stress in mediating the disrupting effect on glycemic control. More specifically, the results of the present data led us to speculate that cadmium causes, as suggested in experimental studies, insulin resistance by boosting the production of oxygen free radicals. However, our study has some limits such as this cross-sectional investigation has been carried out only in male adolescents and, at the present, we do not know whether the same conclusion can be extended to the female gender characterized by a different hormonal status. Furthermore, we lack information on the history of T2D in the family of enrolled adolescents. However, the presence of a control population that suffers from the same bias mitigates these methodological weaknesses.

## 5. Conclusion

This study, for the first time, indicates that cadmium burden alters glycemic control in adolescents and suggests that oxidative stress plays a key role in cadmium-induced insulin resistance that may augment at an older age, the risk of developing metabolic disorder.

## Figures and Tables

**Table 1:** Characteristics of the study population.

	Exposed		Controls		
	Median	IQ range	Median	IQ range	*p*
Age (yrs)	13.1	12.7–13.8	13.0	12.5–13.6	0.326
Weight (kg)	51.0	44.0–63.0	52.0	48.6–56.5	0.623
Height (cm)	158.0	153.5–164.5	158.0	150.0–163.0	0.127
BMI	20.2	18.1–24.6	21.2	19.4–22.9	0.474
Tanner (stage)	3.0	2.0–3.0	3.0	3.0–4.0	<0.001
Testicular volume (ml)	6.2	3.9–8.7	11.3	9.4–15.2	<0.001
Testosterone (nmol/l)	1.0	0.2–1.7	9.5	6.7–15.9	<0.001
Cadmium (*μ*g/L)	0.7	0.3–1.0	0.2	0.0–0.2	<0.001
HOMA-IR	2.3	1.0–4.6	0.9	0.4–1.2	<0.001
TAC *μ*mol/dl	149.6	131.5–160.5	167.4	161.9–171.3	<0.001
MDA *μ*mol/l	7.0	6.0–8.0	3.7	3.1–4.0	<0.001
